# Preoperative glycaemic control and severity of sternal wound infections after cardiac surgery: association of elevated Hemoglobin A1c with deep versus superficial infections

**DOI:** 10.1099/acmi.0.001270.v5

**Published:** 2026-11-12

**Authors:** Maria Khan, Farida Khurram Lalani, Saba Khan, Sheza Batool, Umme Farwa, Shifa Basharat

**Affiliations:** 1 Pathology Department, Peshawar Institute of Cardiology-MTI, Peshawar, Pakistan; 2 Pathology Department, Fazaia Medical College, Air University, Islamabad, Pakistan; 3 Islamic International Medical College, Rawalpindi, Pakistan; 4 Pathology Department, Rehman Medical College, Peshawar, Pakistan

**Keywords:** cardiac surgical procedures, diabetes mellitus, glycaemic control, glycated haemoglobin, preoperative care, surgical wound infection

## Abstract

Sternal wound infections (SWIs), including superficial sternal wound infection (SSWI) and deep sternal wound infection (DSWI), are complications after coronary artery bypass grafting and other cardiac surgeries. Elevated preoperative HbA1c, suggesting poor glycaemic control, impairs wound healing and may escalate postoperative risk. This study evaluates the association between preoperative HbA1c levels and the anatomical severity (superficial versus deep) of SWIs among patients who develop postoperative wound complications. A 24-month retrospective observational study was conducted at a tertiary care cardiac surgery centre. Adult patients (≥18 years) going through elective or urgent isolated cardiac surgery with a preoperative HbA1c measured within 30 days were included. Using consecutive sampling, 133 patients who developed SWIs were identified. DSWI and SSWI were defined according to Centers for Disease Control and Prevention criteria, and logistic regression was employed to evaluate the association between HbA1c categories and the odds of presenting with DSWI rather than SSWI. Among 133 sternal infections, 69.9% were DSWI and 30.1% were SSWI. Diabetic-range HbA1c was common (62.4%) and was associated with a higher proportion of DSWI, occurring in 94% of diabetic-range patients versus 26.5% in normal and 37.5% in pre-diabetic groups χ^2^ = 61.357, *P*<0.001. Univariable logistic regression demonstrated that, among patients who developed a wound infection, those with a diabetic-range HbA1c had significantly higher odds of the infection involving deep tissues rather than remaining superficial (OR 36.40; 95% CI 12.59–105.18; *P*<0.001). Among patients who develop postoperative SWIs, an elevated preoperative HbA1c is strongly associated with deep rather than superficial infection involvement. While this case-only design cannot establish the absolute risk of infection development, preoperative HbA1c serves as a valuable indicator of potential anatomical severity within an infected cohort. Rather than mandating clinical delays, routine preoperative HbA1c assessment should be utilized as a non-invasive risk-stratification tool to identify patients who may benefit from closer perioperative metabolic optimization and extended postoperative surveillance.

## Data Summary

All data generated or analyzed during this study are included in this published article.

## Introduction

Coronary artery bypass grafting (CABG) and other cardiac surgeries may be associated with a serious postoperative complication, sternal wound infections (SWIs). This may lead to increased morbidity, extended hospitalization and high healthcare costs [1–3]. Quite a few risk factors have been identified. Among them, poor glycaemic control has often been shown to impair wound healing and increase susceptibility to infection [4]. Diabetes mellitus (DM) is one of the most observed systemic diseases in surgical patients. In the current guidelines of the American Diabetes Association (ADA), four criteria have been determined for the diagnosis of DM: fasting plasma glucose ≥126 mg dl^−1^; 2 h plasma glucose during oral glucose tolerance test ≥200 mg dl^−1^; a random plasma glucose ≥200 mg dl^−1^; haemoglobin A1c (HbA1c) ≥6.5%. One of these criteria is adequate for diagnosis of DM [5].

Glycated haemoglobin (HbA1c) is a marker of long-term glycaemic status and may be used to assess preoperative metabolic control. Increased HbA1c levels have been linked to undesirable postoperative outcomes, including infection, renal dysfunction and delayed wound healing [6]. The cut-off value of HbA1c is recommended as 6.5% (48 mmol mol^−1^) by ADA in recent guidelines [4]. In Chinese adult patients, three authors found three different values in different trials. Wang *et al.* obtained 6.5% as a result, while Liu *et al.* and Liang *et al.* found 6.3 and 5.9%, respectively [7–9]. On the other hand, do Vale Moreira *et al.* observed the cut-off point as ≥6.8% for Brazilian adult patients in 714 patients [10].

Deep sternal wound infection (DSWI) is the most severe form of SWI. It may often require reoperation, extended antibiotic therapy and aggressive wound management [11]. Many studies have revealed a substantial association between high preoperative HbA1c and increased risk of DSWI. The risk is extremely evident at HbA1c thresholds of 6.5–7.0% [4, 6]. Suggested mechanisms include hyperglycaemia-induced microvascular dysfunction, impaired leucocyte activity and decreased tissue oxygenation, all of which compromise wound healing and increase infection susceptibility.

Superficial sternal wound infections (SSWIs) that involve skin, subcutaneous tissue and superficial fascia also denote a significant cause of postoperative morbidity after CABG. Although less severe as compared to deep infections, SSWIs may result in delayed recovery, extended hospital stays and an increased healthcare burden. Some evidence suggests that elevated preoperative HbA1c may predispose patients to superficial infections also. This may be because of weakened immune function and delayed epithelial repair in the hyperglycaemic state [4, 12]. Duncan *et al.* found that mortality and morbidity after cardiac surgery were significantly reduced when intraoperative hyper-insulinemic normoglycaemia was achieved and concluded that providing exogenous glucose while targeting normoglycaemia may be preferable to simply normalizing glucose concentrations [13].

Data from the E-CABG registry established higher rates of superficial wound infection among patients with HbA1c>7.0% compared to those with HbA1c≤7.0% (7.2% versus 1.6%, *P*<0.001) [12]. Although we do have data related to this, evidence specifically evaluating the association between HbA1c and SSWI remains limited and less consistent across populations. A meta-analysis highlighted that preoperative HbA1c levels were associated with development of mediastinitis, stroke, pneumonia, sepsis, renal failure and mortality after cardiac surgery [14].

The present study aims to delineate the clinical and glycaemic characteristics of patients experiencing sternal wound complications following cardiac surgical interventions. Specifically, it examines whether preoperative HbA1c levels are associated with the anatomical depth of the infection (deep versus superficial) within this infected cohort.

## Methods

This retrospective study investigated the relationship between preoperative glycaemic control, measured by HbA1c levels, and the anatomical severity/depth of SWI among patients who developed SWIs following cardiac surgery. The research was conducted over 24 months at a single tertiary care cardiac surgery centre, utilizing a retrospective, case-only observational design. Because the study cohort is entirely restricted to the 133 adult patients who actively developed a postoperative SWI, the primary outcome of interest is infection severity (DSWI versus SSWI) rather than infection acquisition risk, following isolated elective or urgent cardiac surgery, such as CABG. Crucially, participants had a preoperative HbA1c measurement within 30 days of surgery. Patients with pre-existing active infections or those positive for methicillin resistant *Staphylcoccus aureus* (MRSA) upon admission were excluded. A total of 153 medical charts of patients presenting with sternal wound issues were initially reviewed; 20 cases were excluded due to missing preoperative HbA1c values within 30 days or incomplete 90-day follow-up data, leaving a final analytical cohort of 133 patients. Owing to the retrospective, case-only nature of this institutional audit, a formal prospective sample size calculation based on population incidence was not applicable. Instead, all consecutive eligible cases during the study period were included (consecutive census of eligible cases), enrolling adult patients who developed a confirmed postoperative SWI at our tertiary centre within the 24-month study window. No statistical imputation algorithms were applied.

The study classified SWIs using the criteria established by the Centers for Disease Control and Prevention: SSWI involves skin, subcutaneous tissue and superficial fascia, while DSWI is the most severe form, potentially requiring reoperation and aggressive wound management. The participants were categorized into three groups based on their preoperative HbA1c results: normal, pre-diabetic and diabetic range. Data, including demographics, surgical history and HbA1c values, were collected from electronic medical records. The diagnosis of DSWI was made by a blinded surgical team, and patients were followed for 90 days’ post-discharge. Data completeness was verified via double-entry validation from electronic medical records.

Statistical analyses were performed using IBM SPSS Statistics for Windows, Version 26.0 (Armonk, NY). The primary objective was to evaluate the unadjusted association between preoperative glycaemic status and the severity of SWIs (SSWI versus DSWI). Continuous baseline variables were not formally evaluated due to the nature of the categorical data extraction. A univariable binary logistic regression model was specified using the binary clinical outcome of infection depth (0 = SSWI, 1 = DSWI), as the dependent variable. The categorical independent variable was preoperative glycaemic status, split into three clinical tiers (normal, pre-diabetic and diabetic range). For the binary logistic regression model, the non-diabetic categories (normal and pre-diabetic) were collapsed into a single consolidated reference category (0 = non-diabetic reference, 1 = diabetic-range). This consolidation was mathematically required to mitigate sparse-cell instability in the regression matrix, given that 94.0% of the diabetic-range patients presented with deep tissue involvement.

Due to the retrospective nature of this registry audit, data on major established surgical and clinical confounders, including body mass index, active smoking status, total operative duration, bilateral internal thoracic artery (BITA), harvesting and perioperative intensive insulin protocols, were omitted from the final model due to incomplete documentation across charts. Consequently, the resulting odds ratio (OR) is reported as a crude, unadjusted estimate. The model’s baseline assumptions were checked: the independence of observations was satisfied by the patient selection criteria, and the absence of multicollinearity was guaranteed by the single-predictor nature of the univariable design. Model goodness of fit and performance were evaluated using the likelihood ratio (x^2^=53.180, df=1, *p*<0.001).

The goal of this research was to recommend integrating HbA1c into the standard preoperative assessment to identify high-risk patients who would benefit from metabolic optimization and extended postoperative surveillance.

## Results

The study cohort included 133 adult patients who developed an SWI following isolated elective or urgent cardiac surgery.

Of the total sternal infections, the majority were classified as DSWI, accounting for 69.9% (*n*=93) of cases, while SSWI accounted for 30.1% (*n*=40). The gender distribution showed that 60 females and 73 males developed an infection. The majority of DSWI cases occurred in males (*n*=50), as shown in Table 1. *χ*^2^ = 0.156, *df* = 1, *p*=0.693 . There is no statistically significant association between gender and infection severity (*p*>0.05). The odds of an infection being deep versus superficial do not vary between male and female patients in this cohort.

**Table 1.** Distribution of SWI types by gender

Wound nature/SSI type	Female (*n*)	Male (*n*)	Total frequency (*n*) percentage
DSWI	43 (46.2%)	50 (53.8%)	93 (69.9%)
SSWI	17 (42.5%)	23 (57.5%)	40 (30.1%)
Total	60 (45.1%)	73 (54.9%)	133 (100.0%)

n, number; SSI, surgical site infection.

CABG was the most frequent surgical procedure associated with infection, performed in 100 patients, representing ~75% of the cohort. Among the CABG patients, DSWI was four times more frequent than SSWI (*n*=74 versus *n*=26). Table 2 outlines the distribution of all surgical procedures carried out. To evaluate whether surgical type influenced infection depth, procedures were grouped into a 2×2 comparison of CABG (*n*=100) versus all other pooled non-CABG open sternotomy procedures (*n*=33). A Pearson chi-square test confirmed that surgical procedure type did not demonstrate a statistically significant association with the depth of the subsequent wound infection (x^2^=1.488, df=1, *p*=0.223; Yates' continuity correction x^2^=1.018, *p*=0.313).

**Table 2.** SWI type by cardiac surgery procedure

Surgical procedure type	Wound nature/SSI type		Total
	DSWI, *n*	SSWI, *n*	
CABG	74	26	100 (75.2%)
Aortic valve replacement (AVR)	4	1	5 (3.8%)
Double valve replacement (DVR)	3	3	6 (4.5%)
CABG+mitral valve replacement (MVR)	4	0	4 (3.0%)
Other complex/combined procedures	8	10	18 (13.5%)
Total	93	40	133 (100.0%)

n, number; SSI, surgical site infection.

A substantial majority of the infected cohort exhibited poor long-term glycaemic control prior to surgery: 62.4% (*n*=83) had diabetic-range HbA1c levels. Only 25.6% (*n*=34) had normal HbA1c, and 12% (*n*=16) were in the pre-diabetic range (Table 3).

**Table 3.** Glycaemic status by infection depth

Preoperative glycaemic status	HbA1c threshold	DSWI, *n* (%)	SSWI, *n* (%)	Total patients, *n*
**Normal**	<5.7%	9 (26.5%)	25 (73.5%)	34
**Pre-diabetic**	5.7–6.4%	6 (37.5%)	10 (62.5%)	16
**Diabetic range**	≥6.5%	78 (94.0%)	5 (6.0%)	83
**Total cohort**		**93 (69.9%)**	**40 (30.1%)**	**133**

Evaluation of the data showed a clear gradient in infection depth across HbA1c categories, reflecting a significant association between glycaemic status and the odds of deep versus superficial infection (Table 3). Patients in the diabetic-range HbA1c group developed DSWI at an exceptionally high rate of 94.0% (*n*=78/83), which was ~3.5 times the rate observed in the normal HbA1c group (26.5%).

The time from surgery to the collection of the first positive culture sample revealed that nearly half of the infections were identified late (Table 4). While 47.4% (*n*=63) of cultures were collected in the typical 11–20-day timeframe, a significant proportion, 43.6% (*n*=58), were collected more than 20 days after surgery, highlighting the high frequency of late-onset complications. The data indicate that while deep infections show a higher percentage of late-onset cases (48.4%) compared to superficial cases (32.5%), this temporal distribution shift does not cross the threshold of statistical significance (*p*>0.05).

**Table 4.** Timing of infection post-surgery

Time to first positive culture	DSWI cohort, *n* (%)	SSWI cohort, *n* (%)	Combined frequency, n (%)
**Early onset (1–10 days)**	7 (7.5%)	5 (12.5%)	12 (9.0%)
**Intermediate onset (11–20 days)**	41 (44.1%)	22 (55.0%)	63 (47.4%)
**Late onset (>20 days)**	45 (48.4%)	13 (32.5%)	58 (43.6%)
**Total**	93 (100.0%)	40 (100.0%)	133 (100.0%)

n, number.

Chi-square testing confirmed a highly significant association between preoperative glycaemic status and infection depth. Logistic regression analysis was performed to assess the association of the HbA1c group with odds of deep versus superficial infection. Using the non-diabetic categories (normal/pre-diabetic) as the reference, the model showed that, among patients who developed a wound infection, those in the diabetic-range HbA1c group had significantly higher odds of the infection involving deep tissues (DSWI) rather than remaining superficial (SSWI) (estimate=3.59, z=5.21, *p*<0.001). The estimated OR for DSWI in the diabetic-range HbA1c group was 36.40 (95% CI: 12.59–105.18), and patients with HbA1c levels in the diabetic range exhibited ~36-fold higher odds of the infection involving deep rather than superficial tissues within this infected cohort. The statistical significance of the intercept (*p*=0.005) reflected the baseline odds of DSWI in the non-diabetic reference group.

## Discussion

Our results establish a robust positive association between preoperative HbA1c levels and the proportion/frequency of deep infections of post-surgical SWIs. In this retrospective analysis encompassing 133 patients who developed SWI following cardiac surgery, most cases (69.9%) were classified as the severe form, DSWI, with SSWIs being relatively uncommon. Among the subset of patients presenting with diabetic-range HbA1c (HbA1c≥6.5%), which constituted 62.4% of the infected cohort, the frequency of deep infections was exceptionally high (94%). This finding is in stark contrast to the DSWI rates observed in the normal HbA1c group (26.5%) and the pre-diabetic group (37.5%). This powerful association was statistically confirmed by the $\chi^{2}$=61.357, *p*<0.001. Crucially, the logistic regression model yielded a significantly estimated OR (OR 36.40; 95% CI: 12.59–105.18) for diabetic-range HbA1c associated with deep tissue involvement over superficial involvement. These statistical findings support the hypothesis that poor long-term preoperative glycaemic control is strongly associated with deep tissue involvement over superficial tissue involvement among patients experiencing post-sternotomy wound complications. Our findings align with established literature indicating that elevated preoperative HbA1c is associated with a greater risk of sternal wound complications after cardiac surgery. For instance, a systematic review and meta-analysis by Biancari and Giordano reported that preoperative HbA1c thresholds of 6–7% were associated with an approximately threefold greater risk of DSWI (relative risk ~3.01, 95% CI: 2.32–3.90) [6]. Furthermore, a more recent dose-response analysis by Zhao *et al.*, specifically in CABG patients, found the OR to be 2.67 (95% CI: 2.00–3.58) for elevated HbA1c predicting DSWI, noting a non-linear dose–response relationship where risk increased once HbA1c>5.7% [4]. Our findings demonstrate a strong, progressive increase in the proportion of deep infections across normal, pre-diabetic and diabetic HbA1c categories, aligning with the broader clinical gradients described in prior meta-analyses and systematic reviews. While previous literature primarily evaluates population-wide infection risk, yielding lower relative risk estimates, our case-only analysis specifically evaluates the anatomical severity and depth of infection among patients who already developed postoperative complications. Within our cohort, patients presenting with diabetic-range HbA1c exhibited an exceptionally high proportion of deep tissue involvement compared to the normal and pre-diabetic groups. This distinct concentration of severe presentations highlights the close relationship between poor long-term glycaemic control and aggressive wound complications. In contrast to some previous, more granular studies, our retrospective analysis did not adjust for multiple potential confounders such as obesity, extended operative time, use of BITA versus internal mammary artery (IMA) or intra- and perioperative hyperglycaemia [1–3]. While previous literature often employed thresholds of HbA1c≥6.5% or ≥7.0% [4], our classification into ‘normal’, ‘pre-diabetic’ and ‘diabetic-range’ may have incorporated higher absolute HbA1c values, and the exceedingly high risk observed in the ‘diabetic-range’ group may reflect this pronounced degree of poor long-term glycaemic control. The progressive increase in the proportion of deep infections across the normal, pre-diabetic and diabetic HbA1c categories reflects an observed clinical gradient consistent with broader meta-analytic findings [4]. The robust association between elevated HbA1c and DSWI tissue depth and severity is highly biologically plausible. Chronic hyperglycaemia impairs host immunity by diminishing neutrophil chemotaxis and phagocytosis while causing microvascular disease that restricts oxygen and nutrient delivery to the surgical site. Additionally, it delays tissue repair by reducing collagen synthesis and increasing the non-enzymatic glycation of structural proteins [15–20]. These collective mechanisms significantly increase the risk of microbial colonization, propagation and progression to severe infection. Furthermore, hyperglycaemia often coexists with other comorbidities (e.g. neuropathy and microvascular disease) that inherently predispose patients to surgical site infection [16]. A separate study supports the gradient of risk, finding a significantly increased risk of DSWIs for each unit increase of HbA1c (OR of 5.29 at a threshold of 7.8%) [21]. In the specific context of sternotomy and cardiac surgery, these underlying pathophysiologic risks are exacerbated by specific surgical factors like sternal re-entry, extended operative time, the use of IMAs (especially BITA), which may compromise sternal perfusion, and the presence of mediastinal drains [1, 2]. The fact that most surgeries in our cohort were CABG (75%) suggests the presence of these known high-risk operative features. Clinical Recommendations and Future Research Directions Analysis of the time to infection indicated that a significant proportion of DSWI cases presented late: cultures were taken in 9% within the first 10 days, 47.4% between 11 and 20 days and 43.6% more than 20 days’ post-surgery. This finding underscores the critical necessity for extended post-operative surveillance and prolonged clinical follow-up in high-risk patients (e.g. those with elevated HbA1c). Considering these findings and the strong association between elevated HbA1c and DSWI observed both here and in the literature [4, 6], we present the following clinical recommendations and outline future research priorities: preoperative HbA1c monitoring may be considered as a non-invasive risk-stratification parameter during standard preoperative workups for cardiac surgery patients. For patients with known DM, ensuring an updated HbA1c screening within 3 months prior to elective procedures offers valuable prognostic insights into potential post-surgical complication severity. While our observational, case-only data cannot establish direct causality, the strong clustering of deep infections within the elevated HbA1c tier suggests that preoperative glycaemic evaluation remains a valuable risk-stratification tool. While our observational data demonstrate a distinct clustering of deep tissue involvement within the elevated HbA1c cohort, these descriptive findings are insufficient to infer direct causality or justify absolute mandates for surgical delay. Prospective, randomized controlled clinical trials remain necessary to evaluate whether delaying elective open cardiac procedures for strict metabolic optimization yields a measurable, reproducible reduction in deep sternal wound complications. Because a substantial proportion of DSWI cases manifest well after discharge (44% after 20 days), implementing structured monitoring, extended post-operative surveillance and robust outpatient linkage is critical, especially to support closer perioperative assessment in patients with poorly controlled diabetes. The efficacy of this approach is supported by a recent study that demonstrated tight perioperative glycaemic control significantly reduced the incidence of DSWI in patients with high preoperative HbA1c [22]. The Society of Thoracic Surgeons’ Practice Guideline Series for Blood Glucose Management similarly recommends the routine use of HbA1c tests for preoperative screening [23]. Future research should focus on addressing several methodological and clinical gaps. Prospective cohort studies are needed to estimate odds of deep versus superficial infection and the adjusted risk associated with elevated HbA1c, controlling for operative variables (e.g. surgical complexity and IMA usage) [1, 2].

Randomized or quasi‐experimental trials are crucial to deliberate whether preoperative optimization of HbA1c (e.g. delaying elective surgery and intensified glycaemic control programmes) demonstrably reduces the odds of deep versus superficial infection. More research is needed to identify optimal HbA1c thresholds for defining increased DSWI risk (e.g. 6.5% versus 7% versus 8%) and to determine whether risk profiles differ significantly across distinct surgical populations (CABG versus valve surgery). Studies must be carried out to incorporate additional diabetes-related variables, such as duration of diabetes, presence of diabetes complications (e.g. nephropathy and neuropathy) and glycaemic variability, which may refine risk stratification beyond a single HbA1c value.

The primary strength of this study lies in its focus on a highly specific tertiary care cardiac surgery cohort at a high-volume centre, providing real-world insight into the severe clinical presentation of SWIs. However, these findings must be interpreted alongside distinct limitations: its single-centre retrospective footprint limits geographic generalizability, the case-only design evaluates infection severity rather than overall developmental risk, and the univariable analysis leaves the results exposed to unadjusted residual confounding from critical perioperative variables like obesity, bypass duration and BITA usage.

## Limitations

- The case-only design restricting analysis to severity among infected patients rather than population risk.
- The univariable model and lack of adjustment for major confounders (obesity, smoking, operative duration and BITA).
- Potential misclassification between DSWI and SSWI based on clinical/culture criteria.
- Single-centre retrospective design limiting generalizability.

## Conclusion

This study demonstrates a strong association between elevated preoperative HbA1c levels and the anatomical severity of postoperative SWIs. Among patients who suffer wound complications, a diabetic-range HbA1c is predominantly linked to deep tissue involvement (DSWI) rather than superficial infection (SSWI). While our case-only design limits our capacity to estimate true population-wide depth and severity or risk of development, the clear stratification of severity underscores the clinical utility of preoperative HbA1c in identifying patients prone to more aggressive wound complications. Although the observed effect size in our cohort (OR 36.40) is markedly higher than prior reports, the findings align with established literature, are biologically plausible and demonstrate a clear observed clinical gradient across glycaemic tiers in infection depth across HbA1c categories, strengthening the validity of these results.

Preoperative HbA1c assessment may assist with risk stratification by helping clinicians identify patients who warrant closer perioperative assessment. For those presenting with elevated levels, clinicians could consider intensified monitoring and extended postoperative surveillance, particularly given the observed proportion of late-onset presentations, alongside careful optimization of perioperative management.

## Abbreviations

ADA: American Diabetes Association
BITA: bilateral internal thoracic artery
CABG: coronary artery bypass grafting
DM: diabetes mellitus
DSWI: deep sternal wound infection
HbA1c: haemoglobin A1c
IMA: internal mammary artery
OR: odds ratio
SSWI: superficial sternal wound infection
SWIs: sternal wound infections
